# Near-infrared (NIR) hyperspectral imaging and multivariate image analysis to study growth characteristics and differences between species and strains of members of the genus *Fusarium*

**DOI:** 10.1007/s00216-012-6313-z

**Published:** 2012-08-18

**Authors:** Paul J. Williams, Paul Geladi, Trevor J. Britz, Marena Manley

**Affiliations:** 1 Department of Food Science, Stellenbosch University, Private Bag X1, Matieland (Stellenbosch), 7602 South Africa; 2 Unit of Biomass Technology and Chemistry, Swedish University of Agricultural Sciences, KBC huset, Linnaeus vaeg 6, 901 87 Umeå, Sweden

**Keywords:** Near-infrared hyperspectral imaging, *Fusarium*, PCA, PLS-DA, Classification gradients

## Abstract

Near-infrared (NIR) hyperspectral imaging was used to study three strains of each of three *Fusarium* spp. (*Fusarium subglutinans*, *Fusarium proliferatum* and *Fusarium verticillioides*) inoculated on potato dextrose agar in Petri dishes after either 72 or 96 h of incubation. Multivariate image analysis was used for cleaning the images and for making principal component analysis (PCA) score plots and score images and local partial least squares discriminant analysis (PLS-DA) models. The score images, including all strains, showed how different the strains were from each other. Using classification gradients, it was possible to show the change in mycelium growth over time. Loading line plots for principal component (PC) 1 and PC2 explained variation between the different *Fusarium* spp. as scattering and chemical differences (protein production), respectively. PLS-DA prediction results (including only the most important strain of each species) showed that it was possible to discriminate between species with *F. verticillioides* the least correctly predicted (between 16 and 47 % pixels correctly predicted). For *F. subglutinans*, 78–100 % pixels were correctly predicted depending on the training and test sets used. Similarly, the percentage correctly predicted values of *F. proliferatum* were 60–80 %. Visualisation of the mycelium radial growth in the PCA score images was made possible due to the use of NIR hyperspectral imaging. This is not possible with bulk spectroscopy in the visible or NIR regions.

**Figure:**
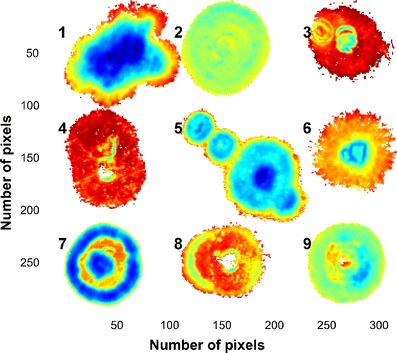
Principal component 1 score image showing differences between colonies. *F. subglutinans* (MRC 0115) are top left followed by *F. proliferatum* (MRC 2301) and *F. verticillioides* (MRC 0826).

Principal component 1 score image showing differences between colonies. *F. subglutinans* (MRC 0115) are top left followed by *F. proliferatum* (MRC 2301) and *F. verticillioides* (MRC 0826).

## Introduction

Fungi are ubiquitous in nature and grow on most substrates under optimal conditions [1]. They are common in tropical and temperate regions and are also found in desert, alpine and arctic areas where harsh climatic conditions prevail. *Fusarium* spp. are mostly regarded as soil-borne fungi where they are abundant and associated with plants as either parasites or saprophytes [1, 2].

Mycotoxin production by *Fusarium* spp. is of primary concern to the food industry. They are known to produce fumonisins, trichothecenes and zearalenones, as well as other minor mycotoxins. Of these, the fumonisins are of particular importance and concern. These toxins are natural contaminants of cereal grains worldwide and are mostly found in maize and products derived from maize. Fumonisins have cancer-promoting activity [3]. Furthermore, *Fusarium verticillioides* strain MRC 0826, isolated from mouldy maize, was shown to cause ELEM in horses, porcine pulmonary edema syndrome in pigs and liver cancer in rats [3–7].

The taxonomy of *Fusarium* spp. has always been a disputable issue [1, 8] and is known as a genus in which it is difficult to distinguish species [9]. The taxonomy has been inundated by varying species concepts, with as few as 9 or well over 1,000 species being recognised [8]. Because of its abundance and the inordinate economic importance of the members of this genus, it is essential to be able to easily and accurately identify the various species.

Conventional identification methods at species level involve plating out on appropriate media, description of colonies (texture, colour and pigment) and microscopic description of conidiogenous cells and conidia [10]. Because variation of important characteristics such as pigmentation and growth rate is often seen within a given species, only well-trained mycologists are able to perform the identification. Other more complex techniques involve molecular techniques such as polymerase chain reaction assays [11, 12], DNA sequencing [10, 13] and mass spectrometry [14, 15]. These techniques are time-consuming, as well as expensive, require specialised technicians, specialised instrumentation and tedious sample preparation. Rapid techniques are thus required for identification and differentiation of fungal species.

Near-infrared (NIR) hyperspectral imaging is an imaging technique in which spectral and spatial information are combined to obtain NIR hyperspectral images [16–19]. NIR hyperspectral images are three-dimensional arrays of the form, **X** (*m × n × λ*), where *m* and *n* are the spatial axes information and the *λ* axis represents the spectral information. The three-dimensional structure of the hypercube requires reorganisation to a two-dimensional matrix to adapt the image for further pre-treatments.

Statistical treatments such as principal component analysis (PCA), an unsupervised classification or dimensionality reduction technique [20], can be applied to the data. It reduces the data to a much smaller number of principal components (PCs) and can be used as an exploratory technique. PC score images and score plots are used interactively to investigate sample images for special features or irregularities in samples. If anomalies are observed during interpretation of cleaned images (irrelevant pixels have been removed), they will most likely be due to relevant variation between samples which could be either chemical or physical. This observed variation can be explained by studying the accompanying PC loading line plots.

Partial least squares (PLS) regression is a powerful regression technique that uses the latent variable approach to find the fundamental relations between two matrices (**X** and **Y**) [21–23]. PLS uses the y-data structure to decompose **X** so that the outcome constitutes an optimal regression vector. Partial least squares discriminant analysis (PLS-DA) operates similarly; however, instead of measured y-data, dummy variables are used which are indicators of groups [24]. This allows for prediction of group membership and thus classification of pixels.

Recently, NIR hyperspectral imaging has been used more frequently in food applications with particular reference to food safety aspects such as detection of fungi on maize kernels [25, 26], early detection of fungal infection on maize [27], detection of chicken heart disease [28], the detection of faecal matter and ingesta on chicken carcasses [29–31], the detection of faecal contamination on apples [32–34] and for the detection of foreign objects in semolina [35] and chicken fillets [36]. Thus far, not much work has been done applying NIR hyperspectral imaging in microbiological studies.

An earlier study investigated NIR hyperspectral imaging as a tool for high-throughput analysis of self-contained microbial identification of test cards for microorganisms of concern in food [37]. In this preliminary work, a NIR chemical imaging system operating in the spectral range 1,000–2,350 nm was used to acquire NIR chemical images of bacterial cells deposited on a ‘card’, containing both the calibration and test samples. Results showed that some bacteria could be identified from differences observed at unique wavelengths and that a standard operating procedure could be developed for a particular ‘card’ to differentiate and hence identify the various organisms it contains using discrete wavelengths. In another study, the detection of *Campylobacter* was investigated with detection accuracies between 97 and 99 % [38]. In this study, an instrument operating in the 400–900-nm wavelength range was used, employing the visible and NIR regions. The disadvantage of this range is that it includes the visible wavelength region, and these models rely on colour as a discriminating tool. A study on the differentiation of toxigenic fungi used a similar wavelength range of 400–1,000 nm and achieved classification accuracy of 97.7 % [39]. Five fungal species were selected, i.e. *Penicillium chrysogenum*, *F. verticillioides*, *Aspergillus parasiticus*, *Trichoderma viride* and *Aspergillus flavus*, and all could be classified using three narrow bands (bandwidth = 2.43 nm) centred at 743, 458 and 541 nm. The last two wavelengths are associated with blue and green, respectively, and resulted in the high accuracy of the classification since four of the five organisms appeared blue/green when cultured on the appropriate medium.

The purpose of the current study was to investigate the use of NIR hyperspectral imaging and multivariate image analysis techniques to differentiate between species and strains of the genus *Fusarium* associated with maize.

## Experimental

### Sample preparation

Three strains of each of three *Fusarium* spp. as shown in Table 1 were kindly supplied by the Department of Plant Pathology, Stellenbosch University, South Africa. The strains were streaked out from a frozen stock solution onto potato dextrose agar (PDA; Merck (Pty) Ltd, Cape Town, South Africa) and incubated at 28 °C in Petri dishes for a total of 96 h.

**Table 1:** Details on the *Fusarium* spp. and respective strains investigated

Species and strains	Isolation locality	Reference
*Fusarium subglutinans*		
MRC 0115	Transkei, Zazulwana, Butterworth	[59]
MRC 2293	USA	[59]
MRC 6194 (KSU E-00990; ATCC 201270; FRC M-3696)	St. Elmo, IL, USA	[60]
*Fusarium proliferatum*		
MRC 2301	California, USA	[59]
MRC 6908	Ghana	na
MRC 7140	Pietermaritzburg, RSA	na
*Fusarium verticillioides*		
MRC 0826	Transkei	[59]
MRC 8267	North Benin	na
MRC 8559 (KSU A-00149; FRC M-3125)	California, USA	[60]

*MRC* Medical Research Council, Tygerberg, South Africa; *ATCC* American Type Culture Collection; *KSU* Kansas State University culture collection, Department of Plant Pathology, Kansas State University, Manhattan, KS, USA; *FRC Fusarium* Research Center, Pennsylvania State University, USA

### NIR hyperspectral imaging system and image collection

Hyperspectral images were acquired with the SisuCHEMA short wave infrared camera (Specim, Spectral Imaging Ltd, Oulu, Finland). The camera comprised an imaging spectrograph coupled to a 2-D array mercury–cadmium–telluride detector. Individual images were acquired within a spectral range of 1,000–2,498 nm at 10 nm resolution, 6.3 nm wavelength intervals and a field-of-view of 100 × 100 mm. Images of the entire Petri dish, without removing the lid, were collected at 3 and 5 days after inoculation. In a few cases where isolates were streaked out in polystyrene Petri dishes, the lid was removed before image collection. Internal dark and white reference standards were imaged prior to each sample.

### Hyperspectral image analysis

Images were analysed using the Evince v.2.5.5 hyperspectral image analysis software package (UmBio AB, Umeå, Sweden) and MATLAB v 7.10 (The MathWorks, MA, USA). The image calibration and correction to absorbance was done automatically in the Evince software package as described in Williams et al. [26].

### Construction of mosaics and image cleaning

Selected individual images within the wavelength range 1,103–2,483 nm were merged to form mosaics. The first mosaic comprised three strains of each of the *Fusarium* spp. The second mosaic contained only the most important strains (MRC 0115, MRC 2301 and MRC 0826) of each species imaged. This included colonies of the same strains in the same Petri dishes, but imaged after 72 and 96 h of incubation, respectively. These strains were selected based on frequency of isolation from contaminated maize [40]. The third mosaic comprised of images of the same strains cultured. The training set was cultured and imaged 18 months prior to the test set. The test set was the same image used as training set in the second mosaic. The fourth mosaic comprised the same training set as the third mosaic (three main strains inoculated on three separate Petri dishes) and the same three strains inoculated in a single Petri dish as the test set.

A PCA model with six components was calculated, on mean centred data, for each of the mosaics (1–4). Using the brushing technique [41, 42], all irrelevant pixels (background, agar, reflection from Petri dish and bad pixels) were removed using score plots and score images of all six principal components (PCs). To ensure efficient cleaning of the images, this cleaning process was repeated five to ten times, and PCA was recalculated after each repetition on the cleaned images.

### Multivariate image analysis

PCA and PLS-DA with species as dummy variables were used as provided in the Evince v.2.5.5 software package. To enable PLS-DA classification with three classes, the samples (pixels) for class one were represented by the row vector (1, 0, 0). Similarly, the samples of class two were represented by (0, 1, 0) and those of class three by (0, 0, 1). The PLS-DA model would thus result in three separate regression coefficients, one for each class for all pixels. These were then used to predict a new set of data which were refolded to form the prediction image, showing the location of the classes.

Different preprocessing methods, i.e. standard normal variate (SNV), multiplicative scatter correction (MSC) and Savitzky–Golay derivatives, were tested to improve models, and the number of components to be used was determined using test sets. Confusion matrices constructed from the test set prediction results were used to evaluate the respective models in terms of percentage pixels not classified, percentage pixels correctly classified and percentage false negatives. The percentage pixels correctly classified were calculated based on total number of pixels in the test set as well as with number of pixels not classified in the test set removed. For the PLS-DA models, prediction images are shown for the training set to illustrate how well the respective models performed. Prediction images for the test sets show the accuracy of the PLS-DA models. PLS-DA models use *R* ^2^ and root mean square error of prediction (RMSEP) per response variable as diagnostics. The optimum number of PLS-DA components was selected based on the number of components that resulted in the highest *R* ^2^, lowest RMSEP and prediction image with best classification of the samples (highest percentage correctly classified pixels).

## Results and discussion

### Hyperspectral image analysis

**Fig. 1:**
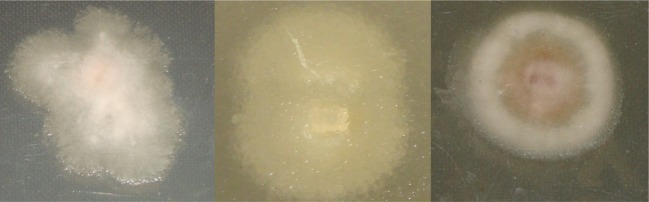
Digital images of mycelium colonies of *F. subglutinans*, *F. proliferatum* and *F. verticillioides* after 72 h incubation at 28 °C

Typical mycelia growth on PDA of *Fusarium subglutinans* (MRC 0115), *Fusarium proliferatum* (MRC 2301) and *F. verticillioides* (MRC 0826) after incubation for 72 h at 28 °C is shown in Fig. 1. A first observation is that the growth is not homogeneous. There is a visible difference between the centre and edges of each colony, but the colonies could otherwise not be visually distinguished based on shape and colour. Visible distinction, between *F. subglutinans* and *F. proliferatum*, is not clear. The non-homogeneous nature of the colonies makes studying the distinction between *Fusarium* spp. an ideal application for NIR hyperspectral imaging and multivariate image analysis.

#### Mosaic 1

**Fig. 2:**
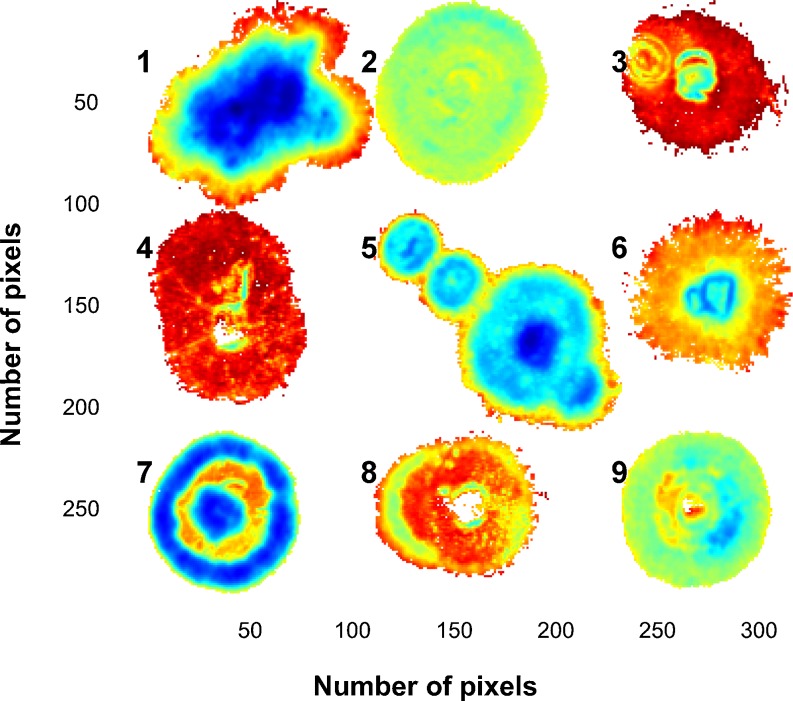
Principal component 1 score image of Mosaic 1 illustrating differences between and within the colonies. Similarities between some of the colonies are also noticeable. Species are shown in rows and strains of the same species within each row. The *left column* comprises the most important strain within each species. *F. subglutinans* (MRC 0115) are *top left* followed by *F. proliferatum* (MRC 2301) and *F. verticillioides* (MRC 0826)

**Fig. 3:**
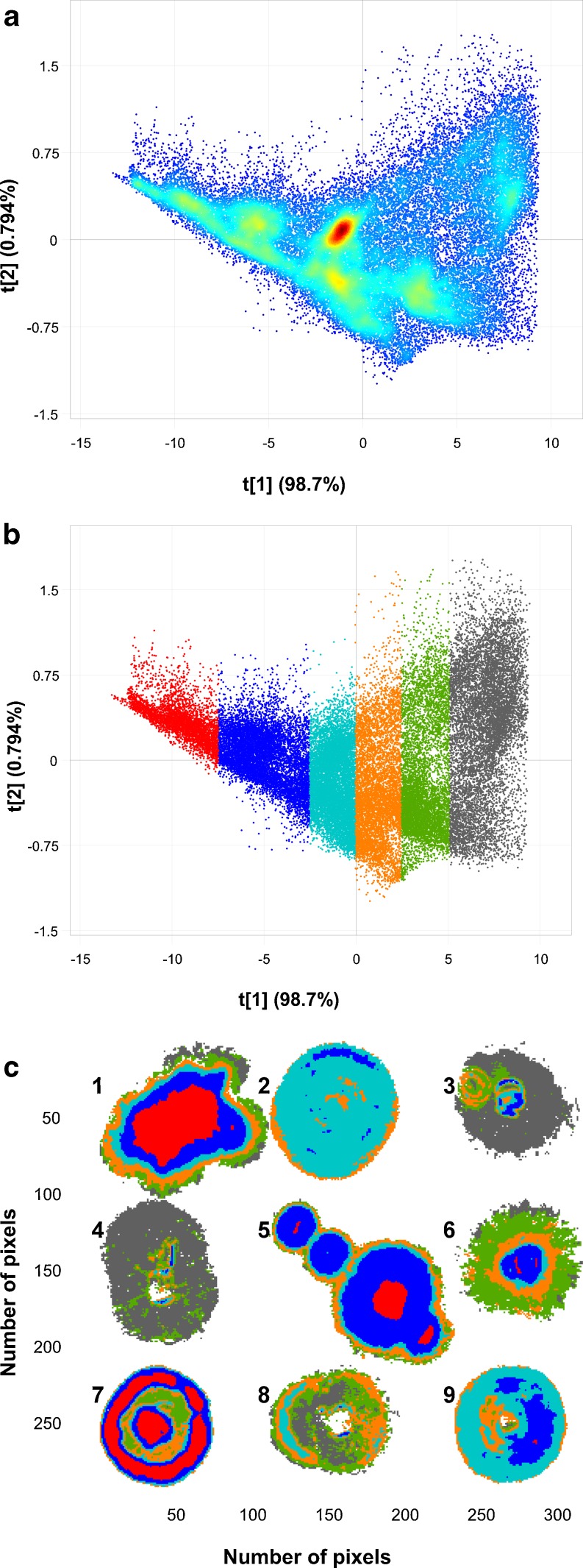
**a** PC1 vs. PC2 scatter plot for Mosaic 1 showing a number of clusters in the PC1 direction, **b** six selected classification gradients in the PC1 direction based on observed clusters and **c** the classification gradients projected onto the score image. Some colonies have a number of clear mycelium growth rings shown as gradient classes (colonies 1, 5, 6 and 7), while others belong mainly to a single gradient class (colony 2) with no apparent difference in mycelium growth over time

By doing a PCA, on mean-centred data, most information on differences between the colonies was found in PC1 [98.7 % sum of squares (SS)] and partly in PC2 (0.79 % SS). PC3 and lower variance PCs showed no relevant information. Figure 2 shows the PC1 score image of Mosaic 1. The mosaic was set up as a nested analysis of variance with the species in rows and the three strains of the same species within each row. The left column comprises the most important strain within each species (most often observed in maize), while the other columns are made up of the more rare strains. Inclusion of the strains less “commonly" associated with maize environments in the mosaic makes distinction between the different species impossible. Preprocessing of the data did not improve these results. Distinction between the three species for the more "common" strains was already clear in the PC1 score image (left column in Fig. 2). Mycelium growth of colonies starts in the centre of the colony with youngest growth toward the edges of the colonies. This phenomenon, referred to as radial growth [43], could clearly be seen within the images of the respective colonies (Fig. 2). This is clearer for some colonies and less clear for others. Temperature plays an important role in the growth, growth rate and the ability of these fungal species to produce mycotoxins [44]. For that reason, all plates were incubated at the same temperature to minimise the variation. In spite of this precaution, it was observed in the score image that all colonies differed markedly. The temperature in this study, i.e. 28 °C, was chosen based on the optimal growth rate for the species involved, reported to be in the range 25–30 °C [44–49]. Selecting an optimum temperature is important when fungi are cultured for a specific purpose such as maximal mycotoxin production. The present study did not have such a purpose, and 28 °C was selected as similar, controlled growth rates were observed at this temperature for all three species. Figure 3a shows obvious density clusters in the score plot, direction of PC1. In the direction of PC2 less clear clustering is seen. Classification gradients [50, 51] were constructed to visualise and understand the meaning and relevance of these clusters (Fig. 3b). Classification gradients were defined in the direction of PC1 by dividing the score value range into six successive groups based on the observed clusters. For interpretation of the groups, the classification plot (Fig 3b), obtained after the groups were selected in the score plot, were projected onto the score image to produce a classification image (Fig. 3c). Figure 3c clearly shows the mycelium radial growth with the older growth of the colonies at the centre of the colony, where the original inoculation was done, and the newer growth toward the edge of the colony showing latest growth after 72 h. These clear differences between the mycelium growth rings further complicated the data classification between species with all the strains included. Because of the different results for the species/strain combinations, growth rings should be studied in more detail by doing a PCA for each colony separately.

### PCA analysis of *Fusarium* spp.

**Fig. 4:**
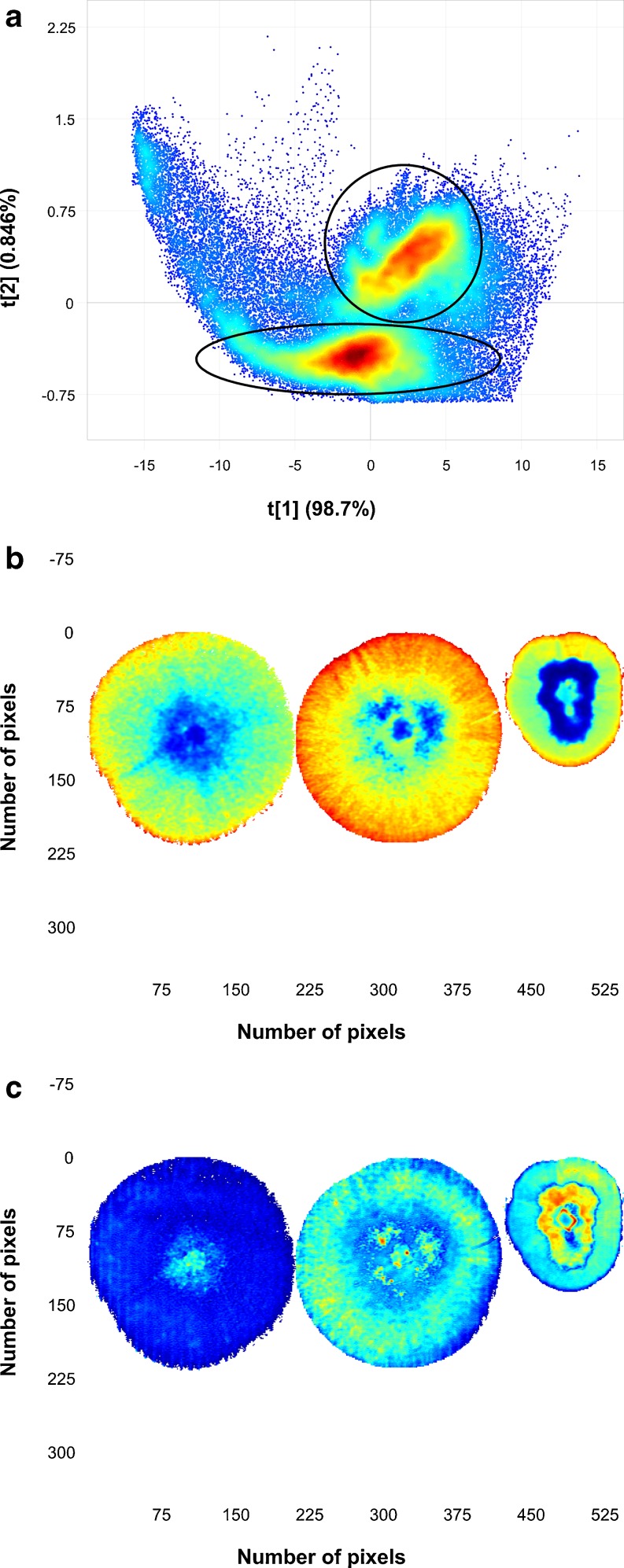

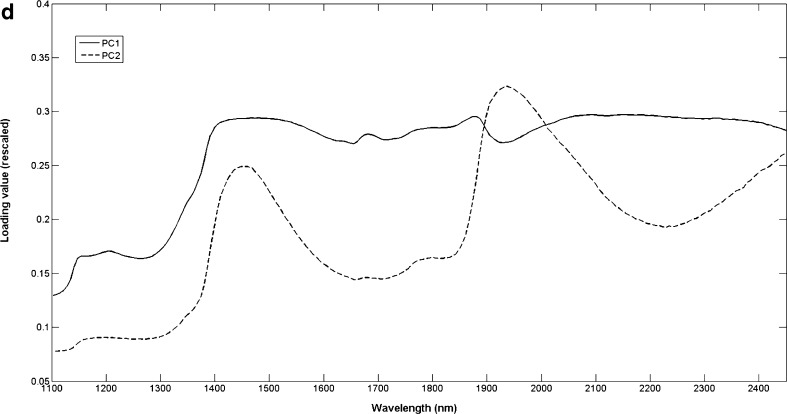
**a** Principal component analysis score plot (PC1 vs. PC2), **b** PC1 score image, **c** PC2 score image and **d** loading line plots of PC1 and PC2 for the most important strain of each of the three *Fusarium* species. *Left to right*: *F. subglutanins*, *F. proliferatum* and *F. verticillioides*

PCA calculated for the most important strain of each of the three *Fusarium* spp., imaged 18 months prior, is shown in the PC1 (98.7 %SS) vs. PC2 (0.846 %SS) score plot (Fig. 4a) and two score images for PC1 (Fig. 4b) and PC2 (Fig 4c), respectively. The score plot showed two clusters in the direction of PC2, and the corresponding score image showed that this was a contrast between *F. subglutinans* and the combination of *F. proliferatum* and *F. verticillioides*. The loading line plot of PC2 explained this contrast due to variation in the N–H stretch first overtone (1,430 nm) and the CONH stretch second overtone (1,918 nm) [52]. These peaks were both positively loaded corresponding with *F. proliferatum* and *F. verticillioides*, both having positive scores in the score plot (Fig. 4d). This means that both these latter species produced more protein during mycelium growth which allowed differentiation from *F. subglutinans*.

The PC1 direction explained the difference between older and newer mycelium growth as can also be seen in the PC1 score image. This was confirmed by means of the loading line plot of PC1, showing a typical shape similar to a mean spectrum which indicated a difference in scattering properties between the different growth stages (Fig. 4d). This difference in scattering properties could also have been caused by the difference in height of the mycelium growth from the centre to the edges of the colonies or due to the presence of spores in the older growth (centre). With NIR hyperspectral imaging, the shape, height and curvature of the sample play an important role. It has been shown that the topography of the sample is explained by the higher variance PCs which accounted for most of the variation in the data [51, 53]. The PC that captured the variation due to chemical differences contributed a low %SS, but still contributed to effective classification. In the present study, the required information to discriminate between the fungal species was explained in PC2 in spite of the low %SS (0.846 %). It is frequently seen in imaging applications that the most relevant PC or PCs are those with a low %SS [54–57]

#### Mosaic 2

To test the effect of growth period of mycelium colonies on PLS-DA classification, Mosaic 2 was made of images of the three *Fusarium* spp. (MRC 0115, MRC 2301 and MRC 0826) scanned at two different times. The training images were collected 72 h after inoculation, while the test set images were collected from the same colonies 96 h after inoculation. The PLS-DA model was calculated with four components on raw, mean-centred data. Dummy (0/1) variables were used as reference data to classify class membership. Preprocessing by SNV, MSC or by Savitzky–Golay derivatives showed no improvement in prediction.

**Fig. 5:**
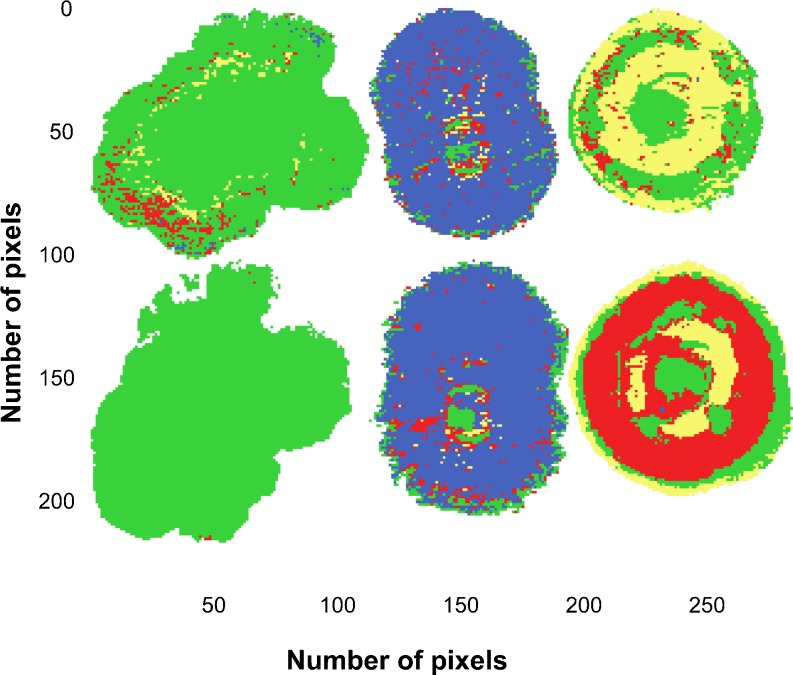
PLS-DA training and test prediction images for Mosaic 2. Classification based on the training data is in the *upper row* for *F. subglutinans*, *F. proliferatum* and *F. verticillioides* (from *left to righ*t; colonies incubated for 72 h), and the *bottom row* is the classification for the test data (the same colonies incubated for 96 h)

The *R* ^2^ of 0.48 for the model for the training data was due to it not being able to clearly distinguish between *F. verticillioides* and *F. subglutinans* as can be seen in Fig. 5. For the same reason, the RMSEP in Table 2 is only acceptable for *F. proliferatum*. The prediction images for *F. subglutinans* (green) and *F. proliferatum* (blue) showed a reasonable number of correctly predicted pixels for both the training and test sets (Fig. 5). For *F. verticilli*oides (yellow), the training image showed misclassification, while the test image showed a large number of pixels not classified (red). Most of the incorrectly predicted pixels in the *F. proliferatum* image were in the centre of the colony, where older mycelium growth was found. *F. verticillioides* showed a number of pixels as not classified (red) with only a few correct predictions. The reason for this high number of not classified pixels could be due to the radial growth that showed marked clustering inside the colony (Figs. 1 and 2c).

**Table 2:** PLS-DA predictions results for Mosaics 2, 3 and 4, showing qualitative classification of *Fusarium* spp. with NIR hyperspectral imaging

	RMSEP	% Not classified	% Correct predictions of only classified pixels	% False negatives	% Correct predictions of total pixels
Mosaic 2					
*F. subglutinans*	0.39	0.1	99.9	0	99.9
*F. proliferatum*	0.25	7.2	86.7	13.3	80.4
*F. verticillioides*	0.45	54.7	44	56	20
Mosaic 3					
*F. subglutinans*	0.58	0.6	98.6	1.4	98.0
*F. proliferatum*	0.44	1.3	66.6	33.4	57.9
*F. verticillioides*	0.40	0.2	16.8	83.2	16.7
Mosaic 4					
*F. subglutinans*	0.32	4.9	80.1	19.9	76.2
*F. proliferatum*	0.45	19.4	86.5	13.5	69.8
*F. verticillioides*	0.46	16.7	39.1	60.9	32.5

From the Evince confusion matrix for the test set, it was calculated that for *F. subglutinans* 99.9 % of the pixels were correctly classified with 0.1 % pixels not classified. For *F. proliferatum*, 86.7 % of the pixels were correctly classified, not taking the non-classified pixels (7.2 %) into consideration. For *F. verticillioides*, the predictions were much poorer with only 44 % correctly classified (54 % not classified pixels not included). More detailed prediction statistics results are shown in Table 2.

#### Mosaic 3

**Fig. 6:**
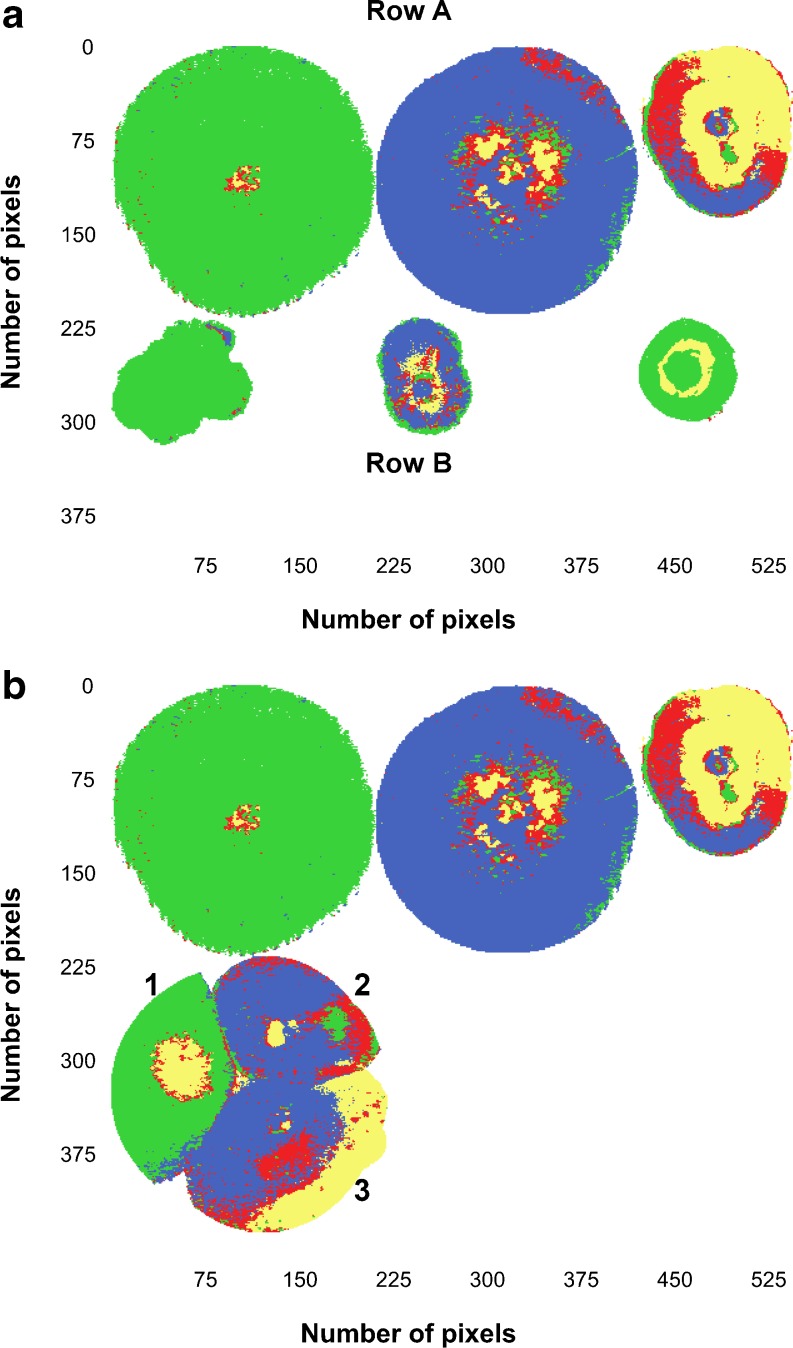
**a** PLS-DA prediction images for Mosaic 3. The *top row* (*row A*) shows images of colonies for *F. subglutinans*, *F. proliferatum* and *F. verticillioides* (from *left to right*) used as training data, and the *bottom row* (*row B*) was the test data. **b** PLS-DA prediction images for Mosaic 4. The test data show the image of the three *Fusarium* spp. inoculated in a single Petri dish (*1 F. subglutinans*, *2 F. proliferatum*, *3 F. verticillioides*)

Images combined in Mosaic 3 comprised two sets of the three *Fusarium* spp., acquired 18 months apart (Fig. 6a). The images in the top row were used as the training set and the images in the bottom row as the test set. This test set is the same that has been used as the training set in Mosaic 2 (as shown in Fig. 5). This means that the model was tested with an independent test set. The PLS-DA model was calculated with four components on mean-centred data. For this mosaic, the *R* ^2^ of 0.66 was reasonable. Again, the RMSEP was lowest for *F. proliferatum*. For the training data (top row, Fig. 6a), *F. subglutinans* showed good predictions. Predictions for *F. proliferatum* were still reasonable, while those for *F. verticillioides* were the worst, although slightly better than the predictions shown in Fig. 5.

For the test set, *F. subglutinans* had a good classification; *F. proliferatum* was slightly worse, but still good, while *F. verticillioides* showed a large number of pixels classified as *F. subglutinans* and only a smaller number of pixels as *F. verticillioides*. From the Evince confusion matrix for the test set, it was calculated that for *F. subglutinans* 98.6 % of the pixels were correctly classified (Table 2). For *F. proliferatum*, 66.6 % were correctly classified, not taking the 13 % pixels not classified into consideration. The predictions for *F. verticillioides* were much poorer (16.8 % correctly classified), although only 0.2 % pixels were not classified. The poor performance of *F. verticillioides* was likely due to inconsistent differences between older and newer growth (as seen in Figs. 1 and 2c). Because of the use of an independent test set, the results were slightly worse than for Mosaic 2. Again, the *F. verticillioides* result was the worst.

#### Mosaic 4

The training data in Mosaic 4 was the same as for Mosaic 3; thus, the same four-component model was used to predict class membership. This time the test set was an image of the three *Fusarium* spp. (MRC 0115, MRC 2301 and MRC 0826) inoculated in a single Petri dish after incubation for 120 h (Fig. 6b). A large number of pixels were not classified for *F. verticillioides* (16.7 %) and even more for *F. proliferatum* (19.4 %). In the previous PLS-DA predictions, the pixels in the *F. subglutinans* image were almost 100 % correctly predicted, while in this mosaic, only 80.1 % of the pixels were correctly predicted with 4.9 % pixels not classified. For *F. verticillioides* and *F. proliferatum*, 39.1 and 86.5 % pixels were correctly predicted (from only classified pixels). For *F. verticillioides*, there was a large number of pixels predicted as false negatives (60.9 %). These pixels were falsely predicted as *F. proliferatum*. A false negative is when the outcome is incorrectly classified as a negative when it is in fact positive. Thus, the majority of the pixels in *F. verticillioides* were classified as not being part of the class, when in fact they were. The PLS-DA prediction results of Mosaics 2 to 4 are compared in Table 2.

Non-classified pixels are not desirable and especially *F. verticillioides* had many of those in two of the mosaics, while *F. subglutinans* rarely had any non-classified pixels. A high proportion of non-classified pixels gives a false impression of correct predictions when the percentage of correct predictions is calculated based only on classified pixels (column three in Table 2). This was clear when the percentage of correct predictions was calculated from the total number of pixels (column 5 in Table 2). The percentage of correctly classified *F. subglutinans* always did best, due to the low percentage of non-classified pixels.

The benefit of using PLS for dimension reduction and discrimination is that it performs better than PCA when within-class variation is higher than between-class variation [58]. In this study the within class variation for *F. verticillioides* seemed to be much higher than for the other species as can be seen in the calibration and prediction images in Figs. 5 and 6. The PLS-DA models, however, failed in classifying *F. verticillioides* efficiently. It seemed that the models could either not classify the pixels of the *F. verticillioides* image as a known class or attempted to predict it as belonging to the *F. subglutinans* or *F. proliferatum* class.

## Conclusion

Using the NIR region, three *Fusarium* spp. could be discriminated from each other with reasonable accuracy by hyperspectral imaging and the use of test sets. Including a number of different strains in the training set complicated identification in PCA score images. This complication was enhanced due to the presence of clear radial growth rings with older growth in the middle and younger growth on the edge of the mycelium colonies. Global diagnostics for the PLS-DA models such as *R* ^2^ and RMSEP serve as a guide of modelling accuracy for images because of the large number of pixels and should be used in conjunction with the prediction image. Occasionally, a model has an unsatisfactory RMSEP, but still shows acceptable prediction results in the prediction image for a large number of pixels. The use of NIR hyperspectral imaging allowed one to visualise radial growth rings in the PCA score images. This would not have been possible with bulk spectroscopy in the visible or NIR regions. Because of this, imaging is far superior to integrating reflectance spectroscopy. An additional advantage of multivariate image analysis is the possibility to interpret PC loading line plots for a possible chemical or physical explanation. Although removing irrelevant pixels from hyperspectral images enhances the ability to detect chemical variation by PCA, inherent physical differences cannot be avoided. Thus, a sound knowledge of the sample(s) is essential for adequate multivariate image analysis. Future research should include experiments on different growth media incubated at variable temperatures and water activities.
